# Statistical design of personalized medicine interventions: The Clarification of Optimal Anticoagulation through Genetics (COAG) trial

**DOI:** 10.1186/1745-6215-11-108

**Published:** 2010-11-17

**Authors:** Benjamin French, Jungnam Joo, Nancy L Geller, Stephen E Kimmel, Yves Rosenberg, Jeffrey L Anderson, Brian F Gage, Julie A Johnson, Jonas H Ellenberg

**Affiliations:** 1 Department of Biostatistics and Epidemiology, University of Pennsylvania School of Medicine, 423 Guardian Drive, Philadelphia, Pennsylvania 19104 USA; 2 Office of Biostatistics Research, National Heart, Lung and Blood Institute, 6701 Rockledge Drive MSC 7913, Bethesda, Maryland 20892 USA; 3 Atherothrombosis and Coronary Artery Disease Branch, National Heart, Lung and Blood Institute, 6701 Rockledge Drive MSC 7956, Bethesda, Maryland 20892 USA; 4 JL Sorenson Heart and Lung Center, Intermountain Medical Center, 5121 S Cottonwood St, Murray, Utah 84107 USA; 5 Division of General Medical Sciences, Washington University School of Medicine, 660 S Euclid Ave, St. Louis, Missouri 63110 USA; 6 Department of Pharmacotherapy and Translational Research, University of Florida College of Pharmacy, Box 100486, Gainesville, Florida 32610 USA

## Abstract

**Background:**

There is currently much interest in pharmacogenetics: determining variation in genes that regulate drug effects, with a particular emphasis on improving drug safety and efficacy. The ability to determine such variation motivates the application of personalized drug therapies that utilize a patient's genetic makeup to determine a safe and effective drug at the correct dose. To ascertain whether a genotype-guided drug therapy improves patient care, a personalized medicine intervention may be evaluated within the framework of a randomized controlled trial. The statistical design of this type of personalized medicine intervention requires special considerations: the distribution of relevant allelic variants in the study population; and whether the pharmacogenetic intervention is equally effective across subpopulations defined by allelic variants.

**Methods:**

The statistical design of the Clarification of Optimal Anticoagulation through Genetics (COAG) trial serves as an illustrative example of a personalized medicine intervention that uses each subject's genotype information. The COAG trial is a multicenter, double blind, randomized clinical trial that will compare two approaches to initiation of warfarin therapy: genotype-guided dosing, the initiation of warfarin therapy based on algorithms using clinical information and genotypes for polymorphisms in *CYP2C9 *and *VKORC1*; and clinical-guided dosing, the initiation of warfarin therapy based on algorithms using only clinical information.

**Results:**

We determine an absolute minimum detectable difference of 5.49% based on an assumed 60% population prevalence of zero or multiple genetic variants in either *CYP2C9 *or *VKORC1 *and an assumed 15% relative effectiveness of genotype-guided warfarin initiation for those with zero or multiple genetic variants. Thus we calculate a sample size of 1238 to achieve a power level of 80% for the primary outcome. We show that reasonable departures from these assumptions may decrease statistical power to 65%.

**Conclusions:**

In a personalized medicine intervention, the minimum detectable difference used in sample size calculations is not a known quantity, but rather an unknown quantity that depends on the genetic makeup of the subjects enrolled. Given the possible sensitivity of sample size and power calculations to these key assumptions, we recommend that they be monitored during the conduct of a personalized medicine intervention.

**Trial Registration:**

clinicaltrials.gov: NCT00839657

## Background

### Personalized Medicine Interventions

The recent availability of lower-cost genetic testing has motivated medical researchers to determine whether patient care and safety is improved by using a patient's genetic information to initiate and manage drug therapy [1]. To evaluate scientific hypotheses regarding a personalized medicine intervention, a randomized clinical trial can be used to contrast outcomes between subjects randomized to receive genotype-guided drug therapy and those randomized to receive an identical therapy without reference to their genetic characteristics [2]. However, because not all subjects may benefit from the pharmacologic intervention due to their genetic makeup, genotype-guided therapy may not benefit the entire study population. Hence, any putative difference between treatment groups will be attenuated, which may adversely impact key components of the statistical design, such as sample size and statistical power. Therefore, the primary statistical challenge of designing a personalized therapy intervention is to accommodate the potential differential effectiveness of genotype-guided therapy across subpopulations defined by allelic variation.

Although interventions that use a subject's clinical factors, gene expression profile, or perhaps other factors can also be considered as personalized medicine, we restrict our attention to interventions that use genotype. In addition, personalized medicine interventions may be evaluated using several different study designs. For example, in a targeted design, frequently used to evaluate genetic-based therapies for cancer, study eligibility may be restricted to a marker-positive subset of the population anticipated to benefit from therapy based on their genetic characteristics [3]. We focus on untargeted designs, such as those that have been used to evaluate genotype-guided dosing of warfarin, in which all subjects are enrolled regardless of their genetic characteristics.

### Genotype-Guided Dosing of Warfarin

Warfarin sodium is the most common oral anticoagulant used for the prevention and treatment of thromboembolism, the formation of a clot in a blood vessel or cardiac chamber that may be carried by the blood stream and obstruct another vessel. Initiation of warfarin therapy is usually based on empiric dosing, which may put patients at an increased risk for either major bleeding complications due to over-anticoagulation or thromboembolic events due to under-anticoagulation. Therefore, initiation of warfarin therapy at an improper dose may be associated with increased costs and higher morbidity [4].

Many patient-specific clinical factors impact warfarin dose-response. In addition, two genes influence warfarin dose: the cytochrome P-450 family 2 subfamily C polypeptide 9 enzyme (*CYP2C9*) gene effects pharmacokinetics, i.e., the effects of the body on the drug; and the vitamin K epoxide reductase complex 1 (*VKORC1*) gene effects pharmacodynamics, i.e., the effects of the drug on the body. Thus, *CYP2C9 *variants alter S-warfarin metabolism [5]; *VKORC1 *variants alter warfarin response [6]. Both *CYP2C9 *and *VKORC1 *have proven useful in algorithms to predict the ultimate maintenance dose for optimal warfarin therapy [7]. However, they have not yet been proven to be beneficial in choosing the initial warfarin dose or to impact clinical outcomes.

The goal of this manuscript is to provide practical guidance on the statistical design of a personalized medicine intervention that uses each subject's genotype information in an untargeted design. The statistical design of the COAG trial serves as an illustrative example. We briefly summarize the clinical rationale and the general study design for the COAG trial. We use power and sample size calculations to illustrate the primary statistical challenge of designing a personalized therapy intervention: to accommodate the potential differential effectiveness of genotype-guided therapy across subpopulations defined by allelic variation. We provide a sensitivity analysis to quantify the extent to which power and sample size calculations may be sensitive to key assumptions required in the statistical design of a personalized medicine intervention. We conclude with general recommendations for the statistical design of personalized medicine interventions.

## Methods

The objective of the COAG trial (clinicaltrials.gov identifier: NCT00839657) is to conduct a multicenter, double blind, randomized clinical trial that compares two approaches to initiation of warfarin therapy:

• *Genotype-guided dosing*, the initiation of warfarin therapy based on algorithms using clinical information and genotypes for polymorphisms in two genes known to influence warfarin response (*CYP2C9 *and *VKORC1*); and

• *Clinical-guided dosing*, the initiation of warfarin therapy based on algorithms using only clinical information.

Both approaches will include a baseline dose-initiation algorithm [8] and a dose-revision algorithm [9] applied after four or five days of warfarin therapy. Subsequent doses will be determined using a standard dose-titration algorithm, which is identical for both groups. By comparing the efficacy of genotype-guided dosing to that of clinical-guided dosing, the COAG trial will determine whether the incremental use of genetic information improves stability of anticoagulation during the early treatment period. Future studies could then determine whether such an improvement leads to significantly reduced costs and lower morbidity.

Eligible subjects will be recruited from at least 12 clinical sites in the United States. Clinical and genotype data will be collected on all subjects. Subjects will be randomized to initiate warfarin therapy either using genotype-guided or clinical-guided dosing. All subjects will receive their warfarin on a standard-of-care schedule. Study investigators, clinicians, and subjects will be blinded to treatment assignment and warfarin dose for the first four weeks of the trial. After four weeks of therapy, subjects will be unblinded to dose and followed for up to an additional five months. The Institutional Review Board of all participating institutions approved the COAG trial. Written informed consent will be obtained from all patients who participate in the trial.

**Figure 1:**
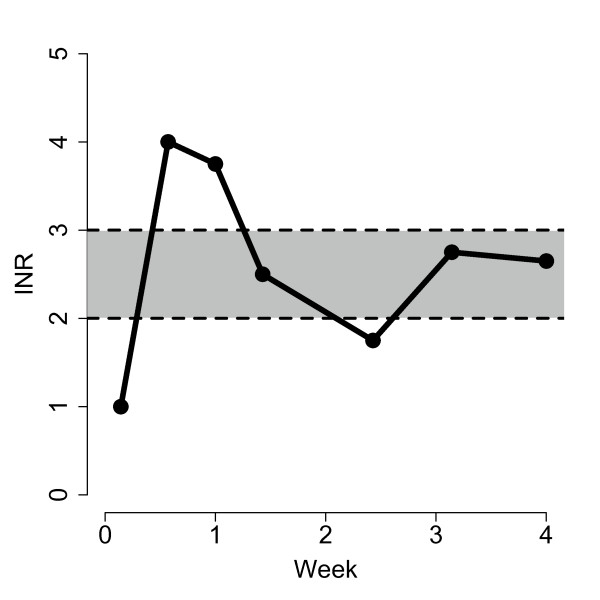
**INR measurements (solid circles) and linear interpolations (solid lines) for a hypothetical subject with 60% of time within the therapeutic INR range (shaded region)**.

The primary outcome of the COAG trial is the percentage of time that participants spend within a therapeutic range for anticoagulation (PTTR) during the first four weeks of therapy. The therapeutic range is defined using the International Normalized Ratio (INR), which reflects the ratio of a patient's prothrombin time to that for a control sample. An INR between 2.0 and 3.0, inclusive, is typically considered to be within the therapeutic range. To calculate the PTTR for each subject, we will use a standard interpolation method that assumes a linear change in INR from one measurement to the next [10]. Figure 1 illustrates the linear interpolation method for a hypothetical subject whose therapeutic INR range is between 2.0 and 3.0, with a corresponding PTTR of 60%.

Analysis of the primary outcome will be by intention-to-treat [11]. It will not be possible for subjects to switch from their assigned treatment group, but there might be crossovers due to the unavailability of genetic information at the time that the initial dose is dispensed. Every attempt will be made to determine a subject's genotype prior to administration of the initial dose. Given recent technologies, same-day genotyping for warfarin is now possible in practice. In the COAG trial, clinical sites are using one of two genotyping platforms; each has a rapid turnaround time. Both platforms have been FDA approved, have high call and concordance rates, very low failure rates, and the ability to genotype the SNPs needed for the selected dosing algorithms.

For those subjects assigned to the genotype-guided dosing group whose genetic information is not available prior to the initial dose, the initial dose will be determined using the clinical dose-initiation algorithm. Once genetic information becomes available, the dose for these subjects will be determined using the genetic dose-initiation and dose-revision algorithms. The genotype-guided dose-initiation algorithm on day one only uses information on *VKORC1 *(not *CYP2C9*) [8]. Therefore, we expect the dose differences on day one to be small relative to the dose differences after the first day. The genotype-guided dose-initiation algorithm on day two, as well as the genotype-guided dose-revision algorithm on days four and five [9], uses information from both *VKORC1 *and *CYP2C9*, so that the availability of genetic information by day two will allow the full use of the subject's genetic information to determine their dose for days two through five. We fully expect genotype information to be available on almost all subjects within 24 hours, and certainly by the time of the dose-revision calculations on days four and five.

### Randomization

To provide balance in treatment assignment within sites, random assignment to either the genotype-guided or clinical-guided dosing group will be stratified by clinical site. Randomization will also be stratified by race (African American versus not, including Caucasian and Asian American) because race is associated with differential predictive ability of dosing algorithms, with lesser accuracy in African Americans [8], and the dosing algorithms used in the trial predict dose differently among African Americans [9]. In addition, African-American race is associated with the prevalence *CYP2C9 *and *VKORC1 *variants and is associated with the prevalence of other genetic variants that influence warfarin dose-response. Finally, some clinical sites may recruit a small number of African Americans due to the demographic makeup of their surrounding community.

We will use a block-randomized procedure to assign the treatment groups. Blocking ensures that there will be a balance in the number of patients in each treatment group within each clinical site. Thus, we will use permuted blocks with block sizes of four and six, randomly chosen, which will minimize any imbalances in treatment group assignment. The RANUNI function in SAS 9.2 will be used to generate the randomization numbers within each site for two strata [12].

### Sample Size and Statistical Power

A critical element of the statistical design of a randomized clinical trial is to determine a sample size so that a statistical test has adequate power to detect a clinically relevant difference in the primary outcome between treatment groups. The parameters considered in the estimation of sample size include: a minimum detectable difference in the primary outcome between groups; an assumed level of significance for the statistical test of the primary outcome; a measure of variability for the primary outcome in the study population; and the percentage of subjects, if any, expected to drop out of the trial. The sample size parameters for a personalized medicine intervention require additional considerations: the distribution of relevant allelic variants in the study population; and whether the intervention is equally effective across subpopulations defined by allelic variants (e.g., if patients with particular genotypes are not expected to benefit from genotype-guided drug therapy, as we illustrate for warfarin). Due to uncertainly in the distribution of allelic variants and uncertainty in the effectiveness of the intervention across subpopulations, careful attention is required in the design of a personalized medicine to ensure that the study will have adequate power to detect a clinically relevant minimum detectable difference.

In the statistical design of the COAG trial, we focused on the difference in the relative effectiveness of genotype-guided across two genetic subpopulations: those with a single genetic variant versus zero or multiple genetic variants in either *CYP2C9 *or *VKORC1*. We viewed the primary outcome of PTTR in each treatment group as a weighted average of PTTR and the corresponding treatment effect (Δ) across subpopulations defined by 1 versus 0, > 1 variants, in which the weights (*w*) are determined by the populations prevalences that sum to 1:

(1) $${\text{PTTR}}_{\text{C}}=w_{1}\times {\text{PTTR}}_{1}+w_{0,>1}\times {\text{PTTR}}_{0,>1;}$$

(2) $${\text{PTTR}}_{\text{G}}=w_{1}\times {\text{PTTR}}_{1}\times \Delta_{1}+w_{0,>1}\times {\text{PTTR}}_{0,>1}\times \Delta_{0,>1;}$$

where PTTR_C _and PTTR_G _denote the PTTR in the clinical-guided and genotype-guided dosing groups, respectively. It is straightforward to generalize this approach to more than two subpopulations of interest. Adding additional terms into the weighted average, given the population prevalence and the anticipated treatment effect in each subpopulation, could accommodate more than two subpopulations. Indeed, this approach is generalizeable to any setting in which treatment effects are expected to differ across any number of subpopulations. Specific assumptions are discussed in the following section.

### Minimum Detectable Difference

We considered the distribution of *CYP2C9 *and *VKORC1 *variants and whether genotype-guided dosing of warfarin is equally effective across groups defined by *CYP2C9 *and *VKORC1 *variants. Current evidence suggests that there will be a subgroup with certain genotypes that will not benefit from genotype-guided dosing [13], most likely because their predicted dose from genotype-guided dosing algorithms will not meaningfully differ from predicted dosing with clinical dosing. We based sample size estimates on the comparison of PTTR between the genotype-guided and clinical-guided dosing groups:

(3) $${\text{PTTR}}_{\text{C}}=0.4\times 73\%+0.6\times 61\%=65.80\%;$$

(4) $${\text{PTTR}}_{\text{G}}=0.4\times 73\%\times 1+0.6\times 61\%\times 1.15=71.29\%;$$

where:

• The proportion in the population who possess a single genetic variant (in either *CYP2C9 *or *VKORC1*) and who possess zero or multiple variants is assumed to be 0.4 and 0.6, respectively;

• A PTTR of 73% and 61% is assumed for those who possess a single genetic variant and for those who possess zero or multiple variants, respectively;

• A 0% relative difference in PTTR is assumed for those with a single genetic variant; and

• A 15% relative difference in PTTR for those with zero or multiple variants is assumed to be a clinically relevant difference between the genotype-guided and clinical-guided dosing groups [14].

We assumed that subjects who possess a single genetic variant (in either *CYP2C9 *or *VKORC1*) would not benefit from clinical-guided dosing because previous data suggest that the genotype-guided algorithm will predict essentially the same dose as the clinical-guided algorithm. These subjects are expected to attain the same PTTR regardless of their treatment assignment and thus attenuate the mean difference in PTTR between the two groups. To wit, the assumed 15% relative difference in PTTR between the genotype-guided and clinical-guided dosing groups is attenuated to an absolute difference of 5.49% (PTTR_G _- PTTR_C_). Therefore, we assumed an overall minimum detectable difference of 5.49% between groups in the full cohort for sample size calculations. If we had ignored the fact that the intervention is not equally effective across subpopulations defined by genetic variants and assumed a minimum detectable difference of 15%, then the trial would have chosen an inadequately small sample size to achieve adequate power.

The assumed proportion of 0.4 who possess a single genetic variant is based on the Couma-Gen trial [13] and the International Warfarin Pharmacogenetics Consortium (IWPC) [15]. We considered the sensitivity of sample size calculations to a range a population proportions. The assumption that those who possess a single genetic variant will not benefit from genotype-guided dosing, while suggested by the Couma-Gen trial, was not supported in another clinical trial in which all patients benefited from dosing based on *CYP2C9 *[16]. In the latter study, the effect of a pharmacogenetic dosing algorithm was similar regardless of the number of *CYP2C9 *variants present. Therefore, we believe that our assumptions are conservative.

### Level of Significance

To determine the level of significance (α) for the statistical test of PTTR between the genotype-guided and clinical-guided dosing groups, we considered an alpha-allocation approach [17-19]. In this approach, a portion (α_A_) of the overall level of significance is used to test the comparison in the full cohort; the remaining portion (α_S_) is used to test the comparison in a pre-defined primary subgroup. The alpha-allocation approach facilitates a traditional primary analysis to assess a statistically significant difference between the treatment groups, as well as a predefined primary subgroup analysis that is not relegated to a secondary analysis, as in a traditional analysis.

We defined the primary subgroup based on subjects whose predicted initial dose employing the genetic and clinical dose-initiation algorithms differs by ≥ 1.0 mg, a factor known at the time of randomization and therefore not a post-randomization selection. We posited that the subgroup of participants with a larger difference between the predicted initial doses should have a larger separation in PTTR between the two groups. If the improvement in PTTR is related to the magnitude of difference in dosing between the genotype-guided and clinical-guided dosing groups, then the primary subgroup comparison should reflect a larger absolute difference than the 5.49% assumed for the full cohort analysis. We assumed that a clinically relevant absolute difference to detect in the primary subgroup is 9.15%, from a PTTR of 61% to 70.15% in Equation (4), reflecting a 15% relative difference.

We selected α_A _= 0.04 for the full cohort analysis and α_S _= 0.01 for the primary subgroup analysis, for an overall type-I error rate of α = 0.05. However, allocating alpha so that sum of α_A _and α_S _is equal to α is a conservative Bonferroni-type correction, which may be unnecessarily conservative if there is a positive correlation between the tests in the full cohort and in the primary subgroup [20,21]. The correlation between the two tests will be obtained under the null hypothesis when the size of the primary subgroup is known. The correlation will then be incorporated to obtain α_S _> α - α_A _given that α_A _is fixed, so that the overall type-I error rate is controlled at α.

Other assumptions in the computation of sample size were the standard deviation of the PTTR in the study population and the percentage of subjects expected to drop out before reaching the primary endpoint. The within-study variability of PTTR in the literature varied across study designs and populations under study. However, there was a reasonable consistency of variability for the genetic-guided and clinical-guided dosing groups in the studies reviewed. We assumed a standard deviation of 25% based on a study of dose-refinement algorithms in which the standard deviation averaged 23% [22]. We also assumed that 10% of subjects would drop out before reaching the primary endpoint and increased the sample size by dividing the calculated sample size by the square of one minus the drop-out rate [23].

### Primary Analysis

The null hypothesis for the primary outcome is that the percent of time that subjects spend within the therapeutic INR range (PTTR) during the first four weeks of therapy is equal between the genotype-guided and clinical-guided dosing groups. We will estimate the difference in mean PTTR between the genotype-guided and clinical-guided dosing groups using a linear regression model, both for the full cohort and for the primary subgroup whose predicted initial dose employing the genetic and clinical dose-initiation algorithms differs by ≥ 1 mg. Inference will be based on a Wald test with a level of significance of 0.05 allocated between the full cohort analysis and the primary subgroup analysis. Because randomization will be stratified by site and race, these variables will be included in the linear regression model. We will perform additional analyses in subgroups defined *a priori *by allelic variation (zero versus a single versus multiple *CYP2C9 *or *VKORC1 *variants) and by race (African American versus not).

Additional genetic factors may be considered in secondary analyses. Specifically, because *CYP2C9 *and *VKORC1 *genotypes may not be the only genetic variants that determine optimal warfarin dosing, it is possible that more variants will be identified during the trial. To adjust for additional genetic factors in secondary analyses, we will include them as covariates in a linear regression model if their prevalence differs between the clinical and genetic groups, and also consider possible interactions with *CYP2C9 *and/or *VKORC1*.

## Results

Table 1 provides the sample size required for the full cohort analysis using a two-sample *t*-test with α_A _= 0.04 (two-sided), assuming various proportions with a single genetic variant (0.4, 0.5, and 0.6), estimates for the standard deviation of PTTR (20%, 25%, and 30%), and power levels (80% and 90%), and drop-out rate (10%). A sample size of 1140 would provide 90% power to detect an absolute difference of 5.49% in the full cohort, given that the proportion with a single genetic variant is 0.4 and the standard deviation is 25%. We selected a sample size of 1238 to protect against departures from the assumed proportion with a single genetic variant, study drop-out rate, and standard deviation of PTTR. For example, if the proportion with a single genetic variant is 0.5 and the standard deviation is 25%, then there is 80% power to detect an absolute difference in PTTR of 4.58%. If the proportion with a single genetic variant is 0.4 and standard deviation is 30%, then there is 80% power to detect the assumed 5.49% absolute difference.

**Table 1:** Sample size estimates for the full cohort analysis; *p *denotes the proportion with a single genetic variant and Δ denotes the corresponding minimum detectable difference

		Standard Deviation of PTTR					
		20%		25%		30%	
		Power		Power		Power	
*p*	Δ	80%	90%	80%	90%	80%	90%
0.4	5.49%	550	730	860	1140	1238	1642
0.5	4.58%	792	1050	1238	1642	1782	2364
0.6	3.67%	1238	1642	1932	2564	2782	3692

A sample size of 1238 provides sufficient power for the primary subgroup analysis using a two-sample *t*-test with α_S _= 0.01 (two-sided). Recall that the size of the primary subgroup is determined by the percentage of subjects whose predicted initial dose employing the genetic and clinical dose-initiation algorithms differs by ≥ 1 mg. If the relative size of the primary subgroup is 50% and the standard deviation of PTTR is 25%, then there is 93.6% power to detect a 9.15% absolute difference. In addition, if the relative size of the primary subgroup is 60% and the standard deviation is 30%, then there is 87.8% power. In fact, the power will be higher because α_S _will be increased according to the correlation between the tests in the full cohort and in the primary subgroup.

### Sensitivity Analysis

In the statistical design of the COAG trial, there was a concern that the genotype-guided and clinical-guided dosing algorithms may not produce sufficiently differentiable doses between the treatment groups, which may lead to an underestimation of the minimum detectable difference in PTTR between groups. We assumed that any difference between the two groups would arise from the subgroup of patients with either zero or multiple genetic variants. (Recall that the assumed relative difference in the genotype-guided dosing group was 15% for those with zero or multiple variants.) For subjects in this allelic subgroup, if the difference between the two algorithm predictions is negligible or clinically irrelevant, then it is reasonable to expect no difference in PTTR. In this case the PTTR for the genotype-guided dosing group can be expressed as:

(5) $${\text{PTTR}}_{\text{G}}=0.4\times 73\%+0.6\times 61\%\times [1+(0.15\times d)],$$

where *d *is the proportion of subjects with zero or multiple genetic variants in whom there is a clinically meaningful difference between the predicted dose determined by the genotype-guided and clinical-guided dosing algorithms. Hence the expected 15% difference would be diluted by a factor *d *and it would be more difficult to detect a clinically relevant difference between groups.

To explore the impact of dilution of the treatment effect, we examined the distribution of the differences between the predicted doses among groups defined by allelic variation in the IWPC cohort [15] and calculated the difference between the rounded predicted doses. An absolute dose difference < 1.0 mg per day was defined as the 'same' predicted dose; an absolute dose difference of ≥ 1.0 mg per day was defined as a 'different' predicted dose. The rationale for the 1.0 mg cut-point is that the average initial dose is 5.0 milligrams; therefore, a 1.0 mg absolute difference represents a clinically relevant 20% difference, on average. Approximately 9% of IWPC participants in the (0, >1) allelic variant group would have received the 'same' initial dose, i.e., *d *= 0.91. With this dilution of the treatment effect, in order to detect an overall effect size of 5.49% in PTTR, the relative effect size in the (0, > 1) group would need to be 16.5%.

Table 2 provides power estimates for the test of the full cohort analysis for a range of diluted treatment effects corresponding to the parameter *d*, the proportion of subjects with zero or multiple genetic variants in whom there is a difference between the predicted doses. There is sufficient power when *d *> 0.9. We are not highly confident in our estimate of how frequently the predicted dose will differ between the two algorithms and therefore have not taken this potential dilution effect into account in our calculations for sample size and power. However, given the potential impact of the dilution effect on the sample size requirements of the study seen in Table 2, we have planned to monitor this factor during the operation of the trial.

**Table 2:** Power estimates for the full cohort analysis in which the treatment effect is diluted; *d *denotes the proportion of subjects with zero or multiple genetic variants in whom there is a difference between the predicted initial doses

*d*	Treatment Effect	Power
0.7	3.84	65%
0.8	4.39	77%
0.9	4.94	86%
1.0 (Undiluted)	5.49	93%

## Discussion

In this manuscript we provided practical guidance on the statistical design of a personalized medicine intervention that uses each subject's genotype information in an untargeted design. We used power and sample size calculations to illustrate the primary statistical challenge of designing this type of personalized therapy intervention: to accommodate the potential differential effectiveness of genotype-guided therapy across subpopulations defined by allelic variation. To determine a minimum detectable difference in PTTR between groups, we assumed that 40% of enrolled subjects would have a single genetic variant and would therefore not benefit from genotype-guided warfarin therapy. Hence, the minimum detectable difference used in sample size calculations is not a known quantity, but rather an unknown quantity that depends on the genetic makeup of the subjects enrolled. In addition, the sample size for the primary subgroup analysis depends on the proportion of subjects whose predicted initial dose employing the genetic and the clinical dose-initiation algorithms differs by ≥ 1.0 mg. Due to the importance of these parameters for adequate sample size and statistical power to detect a clinically meaningful difference, they will be monitored during the course of the trial.

As shown in Table 1, the sample size is sensitive to the standard deviation of PTTR. The Data Safety and Monitoring Board (DSMB) may suggest an 'internal pilot study' in which an estimate of the standard deviation will be obtained using the first half of the observed data and the sample size calculations will be updated based on the new estimate [24,25]. The pre-planned sample size will be assumed to represent a minimum sample size (i.e., the final sample size based on the 'internal pilot study' will not be less than the pre-planned sample size). In this case, the 'internal pilot study' is known as restricted. For restricted designs, the disparity in the type-I error rate in testing the primary hypothesis is negligible [26]. Therefore, it will not be necessary to adjust the type-I error rate of any hypothesis tests regarding the primary outcome. In assessing the need for a sample size increase, data will neither be unblinded nor assessed for the primary outcome. In addition, a sample size adjustment will not impact the overall design of the study. Because the DSMB will not monitor efficacy during the conduct on the COAG trial, there is no conflict between any interim sample size adjustment and interim measures of efficacy.

In our sensitivity analysis, we examined the dilution of the treatment effect due to a clinically irrelevant difference between the predicted doses (employing the genetic and clinical dose-initiation algorithms) for subjects with zero or multiple genetic variants. However, we did not consider the impact of a clinically relevant difference between the predicted doses for subjects with a single variant. In this situation the PTTR for the genotype-guided dosing group can be expressed as:

(6) $${\text{PTTR}}_{\text{G}}=0.4\times 73\%\times [1+(0.15\times d')]+0.6\times 61\%\times [1+(0.15\times d)],$$

where *d*' is the proportion of subjects with a single genetic variant in whom there is a meaningful difference between the predicted doses and *d *is defined in Equation (5). For example, in the IWPC cohort, approximately 26% of subjects with a single genetic variant would have received a 'different' initial dose, i.e., *d*' = 0.26. For these subjects, we expect that there would be a difference in PTTR, which would increase the power of the full cohort analysis. Because we were not highly confident in this estimate, we did not examine the increase in power associated with this allelic subgroup. Therefore, our sensitivity analysis is conservative.

An individual's genetic information could be used prior to randomization to identify subjects who are potentially unresponsive to either drug therapy or the pharmacologic intervention, motivating researchers to decide whether to include or exclude those subjects from the trial [27]. For example, in a targeted design, study eligibility may be restricted to subjects who, based on their genetic characteristics, are predicted to be responsive [28]. By excluding potentially unresponsive individuals, a targeted design will require a smaller sample size to detect a statistically significant effect. Conversely, in a traditional (untargeted) design, particularly of an intervention designed to select dose, subjects for whom genetic-based drug therapy is not effective are eligible, because they would still receive drug treatment regardless of their genetic makeup. For example, subjects in the COAG trial would receive warfarin therapy regardless of their *CYP2C9 *and *VKORC1 *variants. As we have shown with the statistical design of the COAG trial, by including potentially unresponsive subjects, a larger sample size may be required. Cost-benefit considerations regarding the cost of genetic screening for eligibility versus the cost of enrolling potentially unresponsive subjects may be useful to determine which design is more practical in specific applications.

For the COAG trial, we favored including all participants, regardless of their genetic variants. First, the assumption that those who possess a single genetic variant will not benefit from genotype-guided dosing, while suggested by the Couma-Gen trial [13], was not supported in another clinical trial in which all patients benefited from dosing based on *CYP2C9 *[16]. Therefore, if we excluded subjects who may not benefit from genotype-guided dosing, we would be unable to evaluate our assumptions. Second, all subjects are genotyped prior to randomization, so that much of the cost is already incurred in screening. Third, including subjects potentially unresponsive to genotype-guided dosing allows the results of the trial to be more generalizable. That is, if the COAG trial indicates that genotype-guided dosing provides increased efficacy compared to clinical-guided dosing, then it motivates consideration of the policy question of whether all patients prescribed warfarin should be genotyped to predict the drug's efficacy.

We recommend that the statistical design of a personalized medicine intervention that uses each subject's genotype information, within the framework of a randomized clinical trial, consider the distribution of relevant allelic variants in the study population and whether the intervention is equally effective across subpopulations defined by allelic variants. In the statistical design of the COAG trial, we considered the distribution of *CYP2C9 *and *VKORC1 *variants and whether genotype-guided dosing of warfarin therapy would provide an equal improvement in efficacy across populations defined by genetic variants. We assumed that subjects with a single genetic variant would not benefit from genotype-guided dosing, thus attenuating the postulated 15% relative difference between the two treatment groups to a 5.49% absolute difference. In our sample size calculations, if we ignored the fact that the genotype-guided dosing is not equally effective across subpopulations defined by genetic variants and assumed a minimum detectable difference of 15%, then the COAG trial would likely have chosen an inadequately small sample size to achieve adequate power. We also recommend that key assumptions regarding sample size and statistical power be monitored during the conduct of the trial, to inform any requisite increase in the sample size needed to detect a clinically relevant difference in the primary outcome between treatment groups. Further research is required to determine whether an interim sample size adjustment based on the observed proportion of allelic variants increases the type-I error rate.

## Conclusions

In summary, we found that sample size and power calculations may be sensitive to key assumptions required in the design of a personalized medicine intervention: the distribution of relevant allelic variants in the study population; and whether the pharmacogenetic intervention is equally effective across subpopulations defined by allelic variants. Given the novelty of pharmacogenetic research, we recommend that these assumptions be monitored during the conduct of a personalized medicine intervention.

## Competing interests

The authors declare that they have no competing interests.

## Authors' contributions

All authors made substantial contributions to conception and design. BF and JJ drafted the manuscript. All authors revised the manuscript critically for important intellectual content and approved the final manuscript.

## References

[B1] Terra SG Johnson JA Pharmacogenetics, pharmacogenomics, and cardiovascular therapies: The way forward Am J Cardiovasc Drugs 2002 2 287 296 10.2165/00129784-200202050-00001 14727958

[B2] Wang SJ O'Neill RT Hung HM Approaches to evaluation of treatment effect in randomized clinical trials with genomic subset Pharm Stat 2007 6 227 244 10.1002/pst.300 17688238

[B3] Sargent DJ Conley BA Allegra C Collette L Clinical trial designs for predictive marker validation in cancer treatment trials J Clin Oncol 2005 23 2020 2027 10.1200/JCO.2005.01.112 15774793

[B4] Garcia D Regan S Crowther M Hughes RA Hylek EM Warfarin maintenance dosing patterns in clinical practice: Implications for safer anticoagulation in the elderly population Chest 2005 127 2049 2056 10.1378/chest.127.6.2049 15947319

[B5] Sanderson S Emery J Higgins J *CYP2C9 *gene variants, drug dose, and bleeding risk in warfarin-treated patients: A HuGEnet systematic review and meta-analysis Genet Med 2005 7 97 104 10.1097/01.GIM.0000153664.65759.CF 15714076

[B6] Rieder MJ Reiner AP Gage BF Nickerson DA Eby CS McLeod HL Blough DK Thummel KE Veenstra DL Rettie AE Effect of *VKORC1 *haplotypes on transcriptional regulation and warfarin dose N Engl J Med 2005 352 2285 2293 10.1056/NEJMoa044503 15930419

[B7] Schelleman H Chen J Chen Z Christie J Newcomb CW Brensinger CM Price M Whitehead AS Kealey C Thorn CF Samaha FF Kimmel SE Dosing algorithms to predict warfarin maintenance dose in Caucasians and African Americans Clin Pharmacol Ther 2008 84 332 339 10.1038/clpt.2008.101 18596683 PMC2538606

[B8] Gage BF Eby D Johnson JA Deych E Rieder MJ Ridker PM Milligan PE Grice G Lenzini P Rettie AE Aquilante CL Grosso L Marsh S Langaee T Farnett LE Voora D Veenstra DL Glynn RJ Barrett A McLeod HL Use of pharmacogenetic and clinical factors to predict the therapeutic dose of warfarin Clin Pharmacol Ther 2008 84 326 331 10.1038/clpt.2008.10 18305455 PMC2683977

[B9] Lenzini P Wadelius M Kimmel S Anderson JL Jorgensen A Pirmohamed M Caldwell MD Limdi N Burmester JK Dowd MB Angchaisuksiri P Bass AR Chen J Eriksson N Rane A Lindh JD Carlquist JF Horne BD Grice G Milligan PE Eby C Shin J Kim H Kurnik D Stein CM McMillin G Pendleton RC Berg RL Deloukas P Gage BF Integration of genetic, clinical, and laboratory data to refine warfarin dosing Clin Pharmacol Ther 2010 87 572 578 10.1038/clpt.2010.13 20375999 PMC2858245

[B10] Rosendaal FR Cannegieter SC van der Meer FJ Briët E A method to determine the optimal intensity of oral anticoagulant therapy Thromb Haemost 1993 69 236 239 8470047

[B11] Ellenberg JH Armitage P, Colton T Intention-to-treat analysis Encyclopedia of Biostatistics 1998 New York: John Wiley & Sons 2056 2060

[B12] Fishman GS Moore LR A statistical evaluation of multiplicative congruential random number generators with modulus 2^31 ^- 1 J Amer Statist Assoc 1982 77 129 136 10.2307/2287778

[B13] Anderson JL Horne BD Stevens SM Grove AS Barton S Nicholas ZP Kahn SF May HT Samuelson KM Muhlestein JB Carlquist JF Couma-Gen Investigators Randomized trial of genotype-guided versus standard warfarin dosing in patients initiating oral anticoagulation Circulation 2007 116 2563 2570 10.1161/CIRCULATIONAHA.107.737312 17989110

[B14] Dolan G Smith LA Collins S Plumb JM Effect of setting, monitoring intensity and patient experience on anticoagulation control: A systematic review and meta-analysis of the literature Curr Med Res Opin 2008 24 1479 1472 10.1185/030079908X297349 18402715

[B15] The International Warfarin Pharmacogenetics Consortium Estimation of the warfarin dose with clinical and pharmacogenetic data N Engl J Med 2009 360 753 764 10.1056/NEJMoa0809329 19228618 PMC2722908

[B16] Caraco Y Blotnick S Muszkat M *CYP2C9 *genotype-guided warfarin prescribing enhances the efficacy and safety of anticoagulation: A prospective randomized controlled study Clin Pharmacol Ther 2008 83 460 470 10.1038/sj.clpt.6100316 17851566

[B17] Moyé LA P-value interpretation and alpha allocation in clinical trials Ann Epidemiol 1998 8 351 357 10.1016/S1047-2797(98)00003-9 9708870

[B18] Moyé LA Deswal A Trials within trials: Confirmatory subgroup analyses in controlled clinical experiments Control Clin Trials 2001 22 605 619 10.1016/S0197-2456(01)00180-5 11738119

[B19] Coats AJ CAPRICORN: A story of alpha allocation and beta-blockers in left ventricular dysfunction post-MI Int J Cardiol 2001 78 109 113 10.1016/S0167-5273(01)00437-5 11334653

[B20] Alosh M Hugue MF A flexible strategy for testing subgroups and overall population Stat Med 2009 15 3 23 10.1002/sim.3461 18985704

[B21] Joo J Geller NL French B Kimmel SE Rosenberg Y Ellenberg JE Prospective alpha allocation in the Clarification of Optimal Anticoagulation through Genetics (COAG) trial Clin Trials 2010 7 597 604 10.1177/1740774510381285 20693186 PMC3111931

[B22] Lenzini PA Grice GR Milligan PE Dowd MB Subherwal S Deych E Eby CS King CR Porche-Sorbet RM Murphy CV Marchand R Millican EA Barrack RL Clohisy JC Kronquist K Gatchel SK Gage BF Laboratory and clinical outcomes of pharmacogenetic vs. clinical protocols for warfarin initiation in orthopedic patients J Thromb Haemost 2008 6 1655 1662 10.1111/j.1538-7836.2008.03095.x 18662264 PMC2920450

[B23] Lachin JM Introduction to sample size determination and power analysis for clinical trials Control Clin Trials 1981 2 93 113 10.1016/0197-2456(81)90001-5 7273794

[B24] Wittes J Brittain E The role of internal pilot studies in increasing the efficiency of clinical trials Stat Med 1990 9 65 71 10.1002/sim.4780090113 2345839

[B25] Betensky RA Tierney C An examination of methods for sample size recalculation during an experiment Stat Med 1997 16 2587 2598 10.1002/(SICI)1097-0258(19971130)16:22<2587::AID-SIM687>3.0.CO;2-5 9403958

[B26] Wittes J Schabenberger O Zucker D Brittain E Proschan M Internal pilot studies I: Type I error rate of the naive t-test Stat Med 1999 18 3481 91 10.1002/(SICI)1097-0258(19991230)18:24<3481::AID-SIM301>3.0.CO;2-C 10611620

[B27] Simon R The use of genomics in clinical trial design Clin Cancer Res 2008 14 5984 5993 10.1158/1078-0432.CCR-07-4531 18829477

[B28] Simon R Maitouram A Evaluating the efficiency of targeted designs for randomized clinical trials Clin Cancer Res 2004 10 6759 6763 10.1158/1078-0432.CCR-04-0496 15501951

